# Cytokinin modulates the inhibitory effect of shade stress on photosynthesis, antioxidant capacity and hormone homeostasis to regulate the grain yield in wheat

**DOI:** 10.3389/fpls.2024.1498123

**Published:** 2024-11-28

**Authors:** Yongqiang Zhang, Juan Li, Qijiang Xu, Chuanxin Chen, Shihui Nie, Junjie Lei, Liusheng Duan

**Affiliations:** ^1^ College of Agronomy and Biotechnology, China Agricultural University, Beijing, China; ^2^ Research Institute of Grain Crops, Xinjiang Academy of Agricultural Sciences, Urumqi, Xinjiang, China; ^3^ Key Laboratory of Desert-Oasis Crop Physiology, Ecology and Cultivation, Urumqi, Xinjiang, China

**Keywords:** walnut-wheat intercropping, 6-benzyladenine, hormone, wheat yield, physiological mechanism

## Abstract

Agroforestry intercropping is an effective way to optimize land use and ensure food security. However, the physiological mechanism by which the shading of dominant plants inhibits the yield of non-dominant plants in this mode remains to be investigated. A two-year location experiment of walnut-winter wheat intercrop combined with exogenous 6-benzyladenine (6-BA, the first synthetic cytokinin) treatment was conducted to reveal the mechanism of 6-BA in inhibiting wheat growth and yield formation under shade stress by measuring the photosynthetic characteristics, antioxidant capacity, hormone homeostasis of wheat flag leaves and yield. The results showed that compared with far canopy area (FCA), antioxidant enzyme activity [e.g. superoxide dismutase (SOD), peroxidase (POD)], zeatin (ZT) and abscisic acid (ABA) content and photosynthesis of wheat flag leaves were significantly reduced in below canopy area (BCA) treatment during flowering and grain filling stages, thereby inhibiting wheat dry matter accumulation and yield formation. Exogenous 6-BA significantly increased hormone [i.e. indoleacetic acid (IAA), ZT and gibberellin (GA)] levels, antioxidant enzyme activities and photosynthesis in flag leaves, thereby increasing dry matter and yield, especially in the FCA condition. Furthermore, net photosynthetic rate (Pn), stomatal conductance (Gs), intercellular CO_2_ concentration (Ci), activities of ribulose bisphosphate carboxylase (RuBPCase) and phosphoenolpyruvate carboxylase (PEPCase), ABA and ZT concentrations of flag leaves at flowering and filling stages had a significant contribution to yield formation under 6-BA and shade treatments. Overall, cytokinin regulates the inhibitory effects of shade stress on wheat photosynthesis, antioxidant capacity and hormone homeostasis to reduce wheat yield loss.

## Introduction

Agroforestry systems serve as a buffer measure to optimize land use and mitigate the effects of climate change on forest and cereal yields, which plays an important role in addressing the dual pressures of food security challenges from population growth and climate change (Liu et al., 2020; Ivezić et al., 2021; Yang et al., 2023). However, the inhibitory effect of canopy shading on cereal crop yields under this mode is the main concern for farmers (Ai et al., 2021). It is vital to investigate the potential mechanisms by which shading reduces cereal crop yields, and to evaluate appropriate mitigation measures.

Light is the main source of energy for plants to produce assimilates through photosynthesis and also provides a fundamental signal to regulate plant growth and development (Wu et al., 2019). In agroforestry, canopy shading by dominant trees in the canopy causes disadvantaged crops to grow in poor light conditions for long periods of time, significantly reducing the intensity of blue and red light intercepted by disadvantaged crops, as well as photosynthetically active radiation (400-700 nm) (Wolz and DeLucia, 2018; Ullah et al., 2019). Under shade conditions, plants often exhibit shade avoidance responses, including increased cell elongation in various organs (e.g., hypocotyls, stems) and accelerated flowering (Franklin, 2008; Casal, 2013; Ma and Li, 2019). When plants are stressed by prolonged shading, a series of morphological and physiological changes are observed, such as reduction in leaf thickness, damage to chloroplast ultrastructure, decrease in chlorophyll a:b ratio and inhibition of photosystem II activity, which significantly affect photosynthesis and energy homeostasis in plants (Millenaar et al., 2009; Ren et al., 2016). However, the effect of shading on the physiological activity and regulation of growth and development of wheat under intercropping of walnut and winter wheat is still unclear.

Plant hormones are critical in regulating wheat growth under stress (Han et al., 2024), and the role of hormones in the plant response to shade has been reported previously (Stamm and Kumar, 2010). Pons et al. (2001) reported the role of cytokinins in shade avoidance in the response of plants to vertical light intensity gradients in the canopy. Shading significantly reduces cytokinin levels in young, middle and old leaves of soybean (Wu et al., 2017). In leaves, accumulated auxin stimulates the expression of cytokinin oxidase to degrade cytokinins and inhibit leaf growth (Yang and Li, 2017). In the panicles of shade-tolerant rice varieties, most cytokinin pathway genes are up-regulated (Panigrahy et al., 2019). 6-BA is the first artificially synthesised cytokinin (Greene et al., 2016). Spraying with 6-BA increased the number of basal grains and basal lamina, reduced malondialdehyde levels and improves membrane chloroplast structure in maize leaves (Ren et al., 2017). Application of exogenous 6-BA application affected root morphology by altering auxin levels and the expression of key genes involved in auxin synthesis in lateral roots of Malus hupehensis (Mao et al., 2020). However, the physiological effects of 6-BA under shade conditions are still unclear.

In the present study, a two-year study based on the walnut-wheat intercropping model was conducted to explore the potential mechanisms by which shading and cytokinin affect leaf photosynthesis, antioxidant capacity and hormone homeostasis to regulate wheat yield. We investigated two hypotheses: (1) shading inhibits the photosynthetic characteristics of wheat, disrupts endogenous hormone homeostasis and reduces assimilate accumulation; (2) exogenous 6-BA enhances photosynthesis and alleviates yield loss by regulating the physiological activity of wheat flag leaves.

## Materials and methods

### Site description

**Figure 1:**
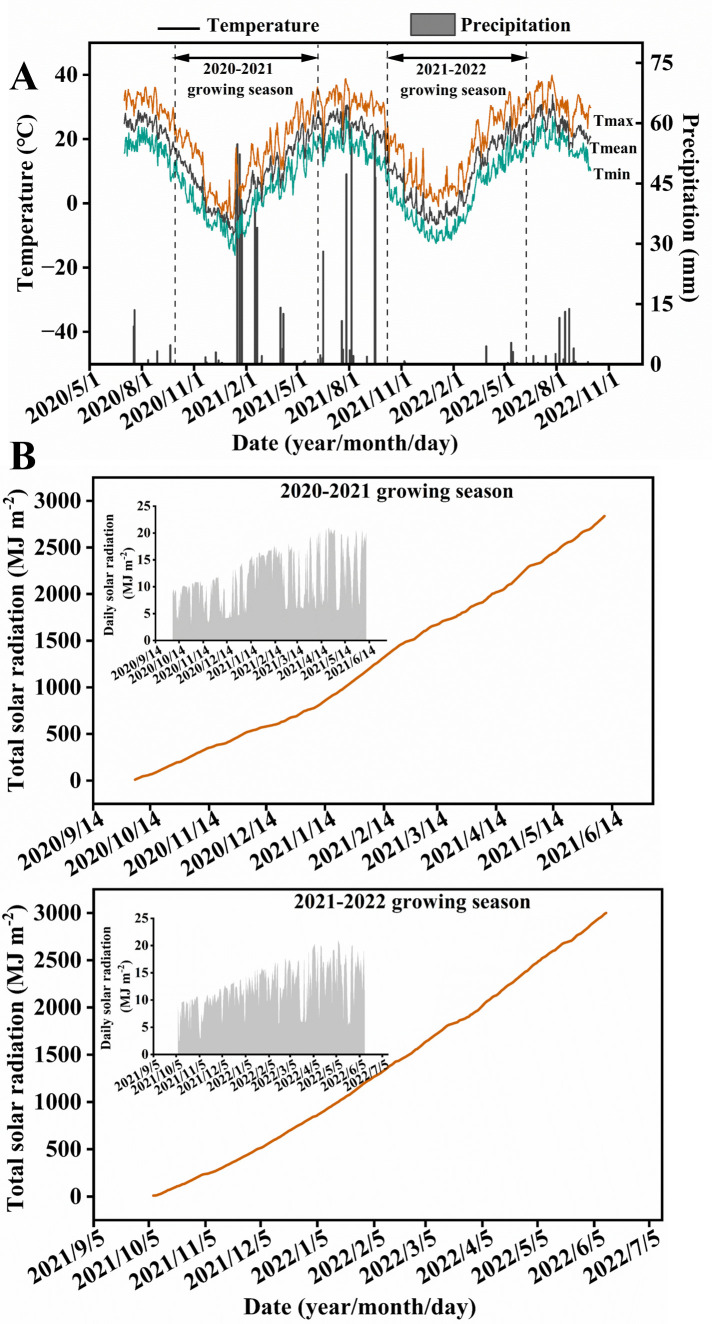
The precipitation, air temperature **(A)** and total/daily solar radiation in each growing season **(B)** of the experimental site in the 2020-2022 growing season. Tmean, Tmax and Tmin represent daily average temperature, daily maximum temperature and daily minimum temperature, respectively.

The experiment was conducted during 2020-2022 in the 5 villages in Ayikule Township, Zepu County, Xinjiang, China (38°18′N, 77°17′E). The altitude ranges from 1215 to 1490 m. The climate is warm temperate continental arid, and the daily average temperature and rainfall during the growth period of winter wheat are shown in **Figure 1A**. The daily solar radiation and total solar radiation for two growing seasons are shown in **Figure 1B**. The former crop was summer soybean, and the soil of the experimental site was sandy loam. The initial soil nutrients of the upper soil layer (0-20 cm) contained 1.5 g kg^-1^ soil organic matter, 0.7 g kg^-1^ total nitrogen, 38.4 mg kg^-1^ alkaline nitrogen, 17.9 mg kg^-1^ available phosphorus, and 102.6 mg kg^-1^ available potassium.

### Experimental design and setup

**Figure 2:**
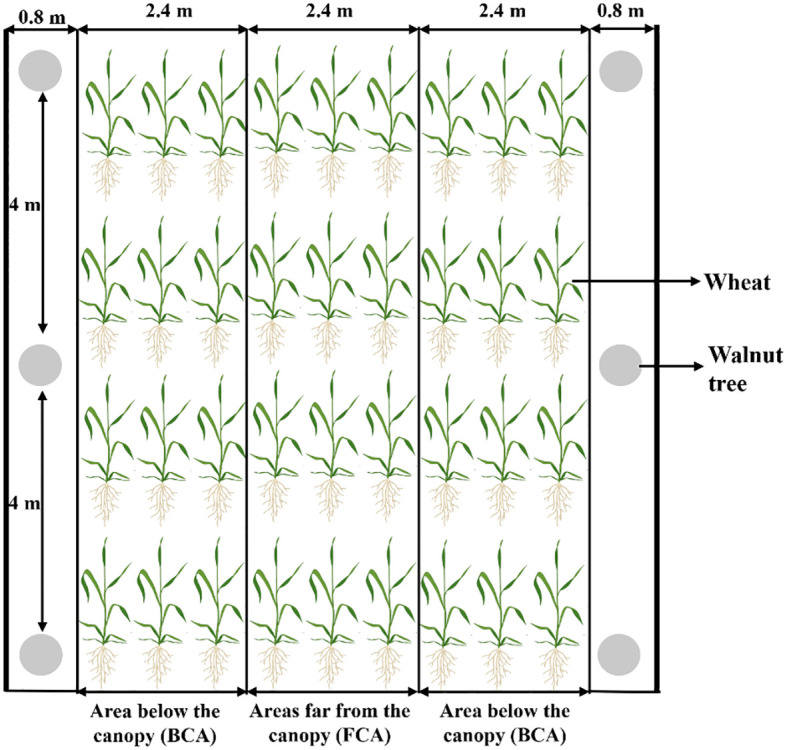
Wheat canopy division under the walnut wheat intercropping pattern.

Field experiment was conducted based on the mode of walnut intercropping with wheat, and a two-factor split-plot experimental design was used in the experiment. The degree of shade corresponding to different canopy areas of the walnut trees were the main factor, including below canopy area (BCA) and far canopy area (FCA) (**Figure 2**); exogenous spraying as secondary factors, including exogenous 6-benzylaminopurine (6-BA) spraying (BCA+6-BA and FCA+6-BA, respectively) and water spraying as control. 6-BA was sprayed once at the booting stage and once at the jointing stage of wheat at a concentration of 60 mg L^-1^, with a spray rate of 750 L ha^-1^, and each treatment was repeated three times. The wheat variety Xindong 60 was sown on October 6 and 8 in 2020, 2021, and harvested on June 10 and 12 in 2021, 2022.The sowing rate was 300 kg ha^-1^ and row spacing of row planting was 15 cm. The walnut tree was 8 years old and evenly spaced north-south with 8 m row spacing and 4 m plant spacing. The width of the walnut-wheat belt was 7.2 m and the minimum distance between the walnut trees and the wheat rows was 1.0 m. The FCA was the area between two corresponding tree trunks located 0.4-2.8 m from the trunk; the BCA referred to the area between two corresponding tree trunks located 2 m from the trunks, as shown in **Figure 2**. The data in the BCA was the average data from two BCA. Before sowing, 150 kg ha^-1^ of urea (containing 46% N) and 300 kg ha^-1^ of diammonium phosphate (containing 46% P_2_O_5_, 18% N) were applied as basic fertilizers. Flood irrigation was applied six times during the whole growing season (overwintering, regreening, jointing, booting, flowering and filling) with a total irrigation volume of 4350-4950 m^3^ ha^-1^. Other cultural practices remained constant in all treatments.

### Plant sampling and measurements

#### Yield and its components

At maturity, select three representative 1 m^2^ to record the number of spikes. Select 15 representative samples of complete wheat spikes to record the number of grains per spike. All wheat spikes are cut and dried naturally, then threshed and 1000 whole grains randomly selected to calculate the weight of 1000 grains. The grains are then weighed and the yield recorded. Three replicates were performed for each plot.

#### Accumulation of assimilates and leaf area index

At the jointing, booting, flowering, filling and maturity stages, two 1-m inner rows of plants were harvested from each plot. The plants were dried at 105 °C for half an hour, and then dried at 75 °C to a constant weight. At the flowering and grain filling stages, leaf area of ten plants was measured manually and LAI was calculated according to Jha (2018).

#### Relative chlorophyll content (SPAD value), photosynthetic and chlorophyll fluorescence parameters

The SPAD values of flag leaves were measured using a SPAD 502 Plus system (Konica Minolta, Japan) at the flowering and filling stages of winter wheat between 9:00 and 11:00 on sunny days. Ten leaves were randomly selected for each treatment. The net photosynthesis rate (Pn), transpiration rate (Tr), stomatal conductance (Gs), and intercellular carbon dioxide concentration (Ci) of flag leaves were determined by a Li-6400XT system (Li-COR, USA) at the flowering and filling stages of winter wheat during 10:30-12:00 am on sunny days, and six leaves were selected at random to measure for each plot. The light intensity was maintained at 1700 μmol·m^-2^·s^-1^. The ambient CO_2_ concentration was measured to be 380 μmol mol^-1^. At the flowering and filling stages, the maximum fluorescence (Fm’) and steady-state fluorescence (Fs) under natural light conditions, as well as the initial fluorescence and maximum fluorescence of the leaves after 60 min of dark adaptation of six flag leaves randomly selected from each treatment, were measured using the Pocket PEA plant efficiency analyzer. Then, the maximum light energy conversion efficiency (Fv/Fm) and quantum yield (Φ psII) of photosystem II were calculated according to Wang et al. (2015).

#### Activities of ribulose bisphosphate carboxylase (RuBPCase) and phosphoenolpyruvate carboxylase (PEPCase)

The activities of RuBPCase (EC 4.1.1.39) and PEPCase (EC 4.1.1.31) of flag leaves at flowering and filling stages were measured by detecting the rate of decrease of NADH at 340nm using enzyme-linked immunosorbent assay kits from Geruisi Biotechnology Co., Ltd. (Suzhou, China) according to the manufacturer’s instructions.

#### Activities of antioxidative enzymes

Superoxide dismutase (SOD; EC 1.15.1.1) activity was assayed by monitoring the inhibition of photochemical reduction of nitro blue tetrazolium (NBT) at 550 nm, and catalase (CAT; EC 1.11.1.6) activity was assayed by measuring the disappearance of H_2_O_2_ at 240 nm (Jiang and Zhang, 2002). Peroxidase (POD) activity was determined by the method of Maehly and Chance (1954), in which guaiacol was converted to tetraguaiacol and monitored at 470 nm (Jiang et al., 2020). Ascorbate peroxidase (APX; EC 1.11.1.11) activity was determined by the decrease in absorbance at 290 nm according to Sharma and Dubey (2004). Glutathione reductase (GR; EC 1.6.4.2) activity was calculated by measuring the rate of decrease in absorbance at 340nm to determine the NADPH dehydrogenation rate (Wang et al., 2014).

#### Endogenous hormone content

The extraction, purification and determination of zeatin (ZT), gibberellin (GA), indole-3-acetic acid (IAA) and abscisic acid (ABA) were performed according to the methods of Lv et al. (2017) and Yang et al. (2001). Specifically, fresh samples (0.5 g) were homogenised with 5 mL of 80% (v/v) methanol containing 1 mmol L^-1^ butylated hydroxytoluene (BHT). The extraction solution was passed through Chromosep C18 columns, the fractions were vacuole-dried at 40 °C and dissolved in 1 mL phosphate-buffered saline (PBS) containing 0.1% (v/v) Tween 20 and 0.1% (w/v) gelatin (pH 7.5). Quantification of ZR, GA, IAA and ABA were performed by enzyme-linked immunosorbent assay.

### Statistical analysis and graphing

The data were subjected to analysis of variance (ANOVA) using SPSS Statistics 26.0 (Chicago, USA). The differences between means were compared using the least significant difference (LSD) test with a *P* value of < 0.05. The random forest model analysis to rank the contribution of physiological indices at flowering and filling stage to yield was performed using the “ randomForest “ and “ rfPermute “ packages in R. Graphs were generated using Origin 2024b and Microsoft PowerPoint 2016 (Microsoft, Redmond, USA).

## Results

### Dry matter weight, leaf area index (LAI) and yield

**Figure 3:**
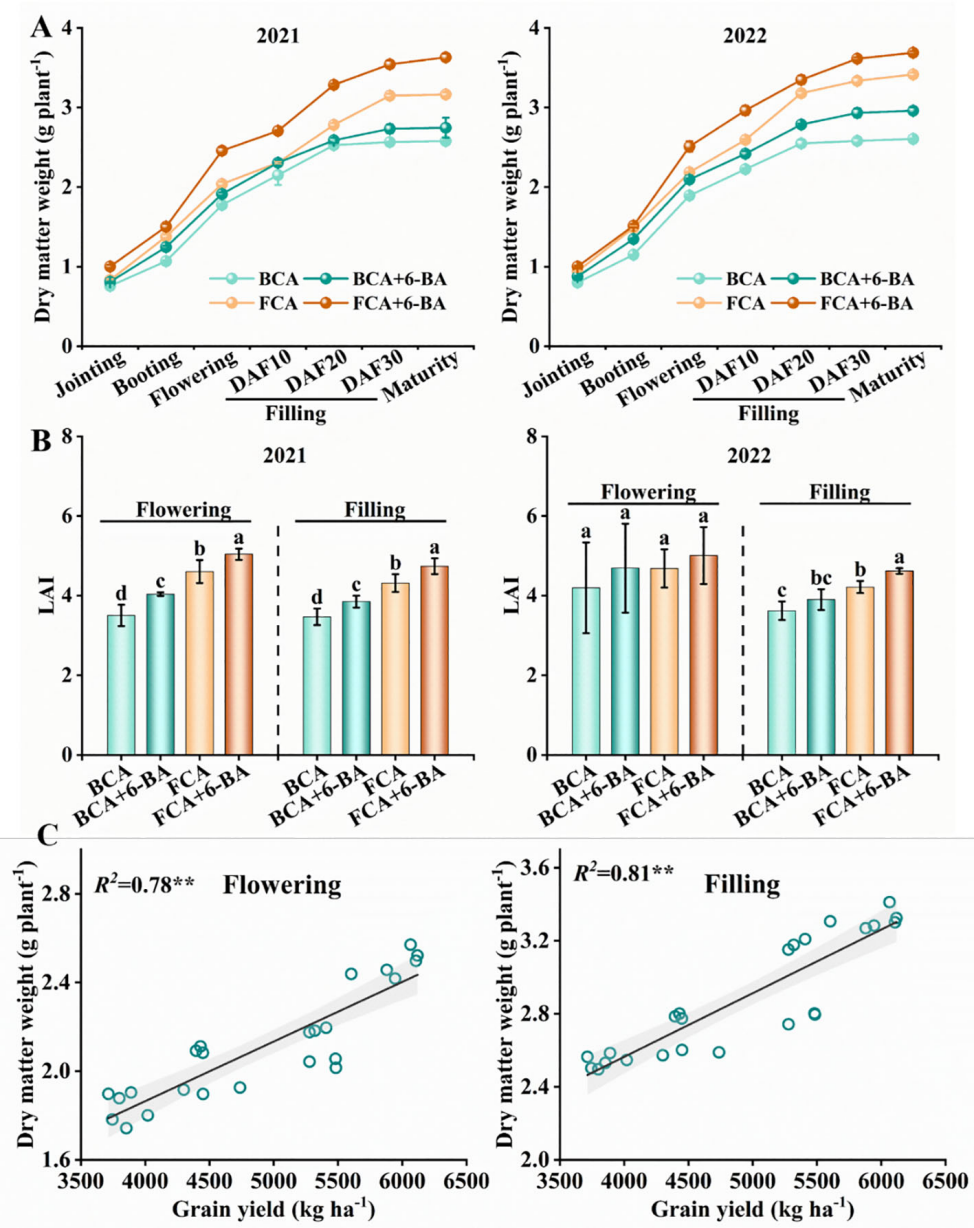
Dry matter accumulation **(A)**, LAI **(B)** and linear fit between dry matter and yield **(C)** in wheat under shade and exogenous 6-BA treatments. LAI, leaf area index; BCA, below canopy area; FCA, far canopy area; BCA+6-BA, exogenous spraying of 6-BA under BCA; FCA+6-BA, exogenous spraying of 6-BA under FCA. Values in **(B)** are means ± SD (n=3). Different lowercase letters denote significant differences (ANOVA, Fisher’s least significant difference test, *P*<0.05). ** indicate significance at the *P* < 0.01 level.

In the walnut-wheat intercrop, shade stress significantly inhibited wheat growth (**Figure 3**). Specifically, the dry matter weight of single wheat stems was significantly lower in the BCA treatment compared to FCA, especially at maturity (**Figure 3A**). Meanwhile, compared to the FCA treatment, the LAI of wheat in the BCA treatment was reduced by 10.4%-23.9% at the flowering and filling stages (**Figure 3B**). Interestingly, wheat dry matter accumulation and LAI (except for 2022) from flowering to filling were increased by exogenous 6-BA, both under BCA and FCA conditions. Correspondingly, affected by canopy shading of walnut trees, the number of effective spikes decreased by 10.81%-11.64%, number of grains per spikes decreased by 7.9%-10.1%, thousand grain weight decreased by 8.1%-10.7%, and grain yield of wheat decreased by 26.6%-27.0% in 2 years in BCA treatment compared with FCA. In the BCA and FCA treatments, the number of effective spikes increased by 1.39% to 3.44%, the number of grains per spike by 4.4% to 9.18%, the thousand grain weight by 2.3% to 5.5% and the yield by 10.4% to 16.5% when exogenous 6-BA was applied compared to spray water (**Table 1**). There was a significant positive correlation between wheat dry matter weight at flowering and grain filling and yield at maturity, with R2 values of 0.78 and 0.81, respectively (**Figure 3C**).

**Table 1:** Wheat yield and its components during two growing seasons.

Years	Treatments	Spikes number (×10^4^ ha^-1^)	Grains number per spike	1000-grains weight (g)	Yield (kg ha^-1^)
2021	BCA	548.84d	28.55c	29.78d	3872.18d
	BCA+6-BA	556.49c	30.98b	31.41c	4494.54c
	FCA	613.54b	31.62ab	33.62b	5414.64b
	FCA+6-BA	625.72a	33.00a	34.88a	5977.90a
2022	BCA	541.56d	28.22d	29.96b	3800.75d
	BCA+6-BA	560.20c	30.81c	30.89b	4426.16c
	FCA	614.75b	31.95b	32.73a	5335.21b
	FCA+6-BA	632.19a	33.72a	33.49a	5926.69a
ANOVA	Years (Y)	ns	ns	*	ns
	Treatments (T)	**	**	**	**
	Y×T	**	ns	ns	ns

BCA and FCA represent the area below the canopy and area far from the canopy, respectively; BCA+6-BA and FCA+6-BA represent the exogenous 6-benzylaminopurine (6-BA) under BCA treatment and FCA treatment, respectively. Different lowercase letters denote significant differences at the 0.05 level. * and ** indicate significance at the *P* < 0.05 and 0.01 levels, respectively; ns, no significant difference.

### Photosynthetic characteristics

**Figure 4:**
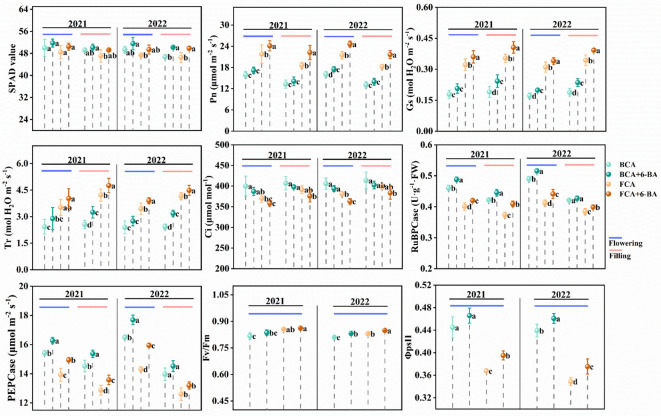
Effect of shading and exogenous 6-BA on SPAD, photosynthetic parameters and fluorescence parameters of wheat flag leaves. SPAD, relative chlorophyll content; Pn, net photosynthetic rate; Tr, Transpiration rate; Gs, Stomatal conductance; Ci, intercellular CO_2_ concentration; RuBPCase, activity of ribulose bisphosphate carboxylase; PEPCase, activity of phosphoenolpyruvate carboxylase; Fv/Fm, maximum light energy conversion efficiency; Φ psII, quantum yield. BCA, below canopy area; FCA, far canopy area; BCA+6-BA, exogenous spraying of 6-BA under BCA; FCA+6-BA, exogenous spraying of 6-BA under FCA. Values are means ± SD (n=3). Different lowercase letters denote significant differences (ANOVA, Fisher’s least significant difference test, *P*<0.05).

The photosynthetic characteristics of wheat flag leaves were significant differences during the flowering and grain filling stages in different canopy regions. In particular, the SPAD value of wheat flag leaf was significantly higher in the BCA than in the FCA (**Figure 4**). The SPAD value of flag leaves was significantly increased by exogenous 6-BA under BCA and FCA conditions. Meanwhile, Pn, Gs and Tr of winter wheat flag leaves were significantly reduced in BCA compared with FCA, while Ci was significantly increased (**Figure 4**). The Pn, Tr and Gs of wheat flag leaves during the flowering and grain filling stages were significantly increased under exogenous 6-BA, while the Ci of flag leaves was reduced. Further analysis of photosynthetic physiological parameters revealed that the activity of RuBPCase and PEPCase in wheat flag leaves gradually decreased from flowering to grain filling stage (**Figure 4**). The RuBPCase and PEPCase activities of wheat flag leaves in BCA were higher than those in FCA, and the change pattern of 2-year experimental results between treatments was consistent; the RuBPCase and PEPCase activities of wheat flag leaves in BCA and FCA under the intercropping mode of walnut wheat were increased to different degrees by spraying exogenous 6-BA. During the two years, the Fv/Fm and φ II of wheat flag leaves in the flowering period showed similar trends (**Figure 4**).

### Enzymatic antioxidant system

**Figure 5:**
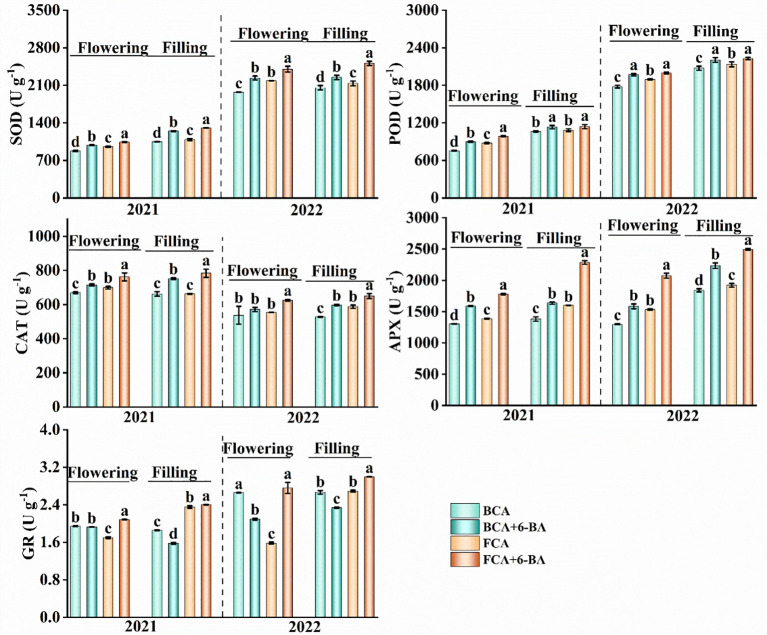
Effect of shade and exogenous 6-BA on antioxidant enzyme activities of wheat flag leaves. SOD, superoxide dismutase activity; POD, peroxidase activity; CAT, catalase activity; APX, ascorbate peroxidase activity; GR, glutathione reductase. BCA, below canopy area; FCA, far canopy area; BCA+6-BA, exogenous spraying of 6-BA under BCA; FCA+6-BA, exogenous spraying of 6-BA under FCA. Values are means ± SD (n=3). Different lowercase letters denote significant differences (ANOVA, Fisher’s least significant difference test, *P*<0.05).

**Figure 6:**
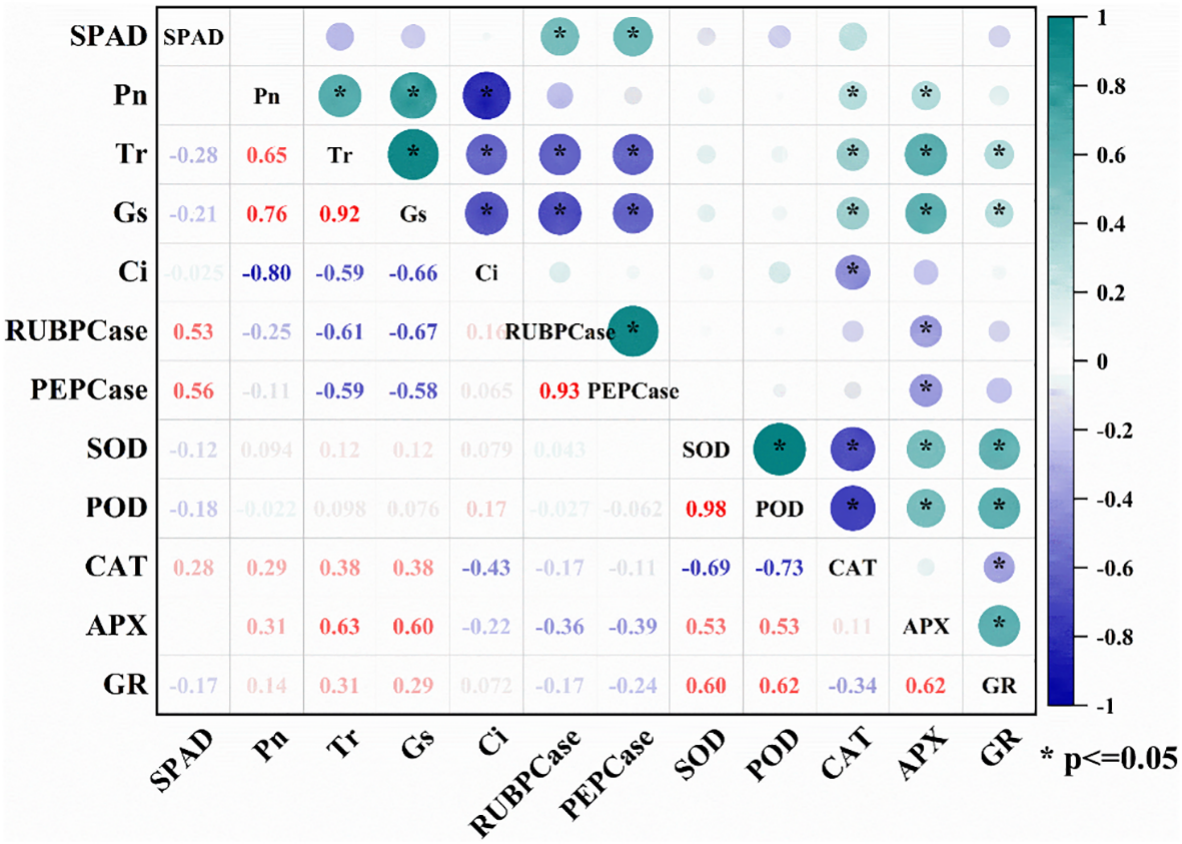
Correlation analysis between antioxidant enzyme activities and photosynthetic parameters in wheat flag leaves at flowering and filling stages. SPAD, relative chlorophyll content; Pn, net photosynthetic rate; Tr, Transpiration rate; Gs, Stomatal conductance; Ci, intercellular CO_2_ concentration; RuBPCase, activity of ribulose bisphosphate carboxylase; PEPCase, activity of phosphoenolpyruvate carboxylase; SOD, superoxide dismutase activity; POD, peroxidase activity; CAT, catalase activity; APX, ascorbate peroxidase activity; GR, glutathione reductase. * indicate significance at the *P*<0.05.

During the flowering and filling stages, the activities of SOD, POD, CAT and APX in wheat flag leaves were significantly lower in BCA than in FCA (**Figure 5**). The activity of GR in the flowering period was higher in BCA than in FCA, but during the grain filling period was opposite. Compare with spraying water, the activities of SOD, POD, CAT and APX in wheat leaves were significantly increased after spraying 6-BA under intercropping mode, especially under FCA. Moreover, the activity of GR was significantly increased by exogenous 6-BA under FCA. Therefore, exogenous 6-BA enhanced the antioxidant capacity of wheat flag leaf under shade stress caused by walnut-wheat intercrop. There was also a highly significant positive correlation between APX and GR activity and Pn, Tr and Gs (**Figure 6**).

### Endogenous hormone homeostasis

**Figure 7:**
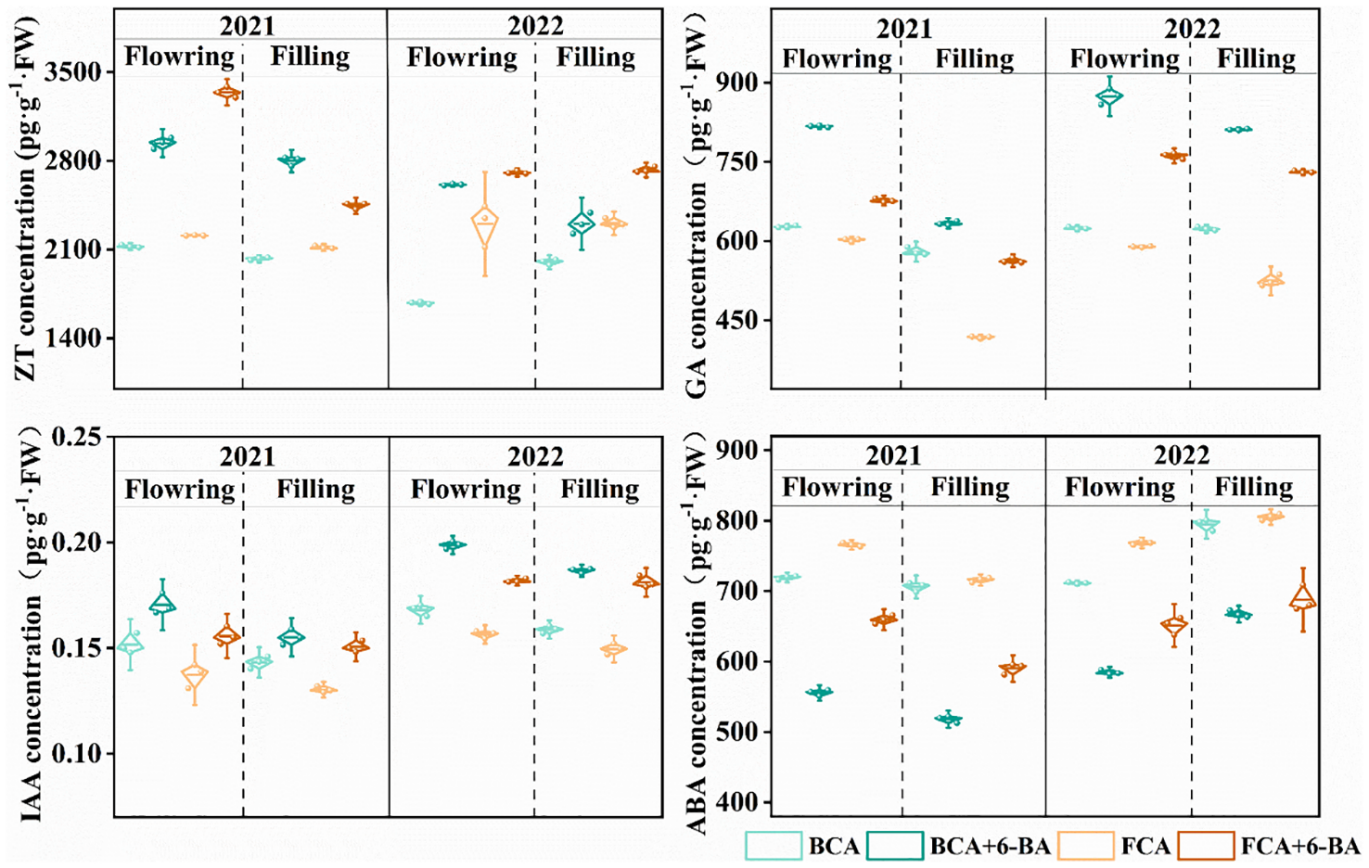
Effect of shade and exogenous 6-BA on endogenous hormone levels in wheat. ZT, zeatin; ABA, abscisic acid; GA, gibberellin; IAA, indoleacetic acid. BCA, below canopy area; FCA, far canopy area; BCA+6-BA, exogenous spraying of 6-BA under BCA; FCA+6-BA, exogenous spraying of 6-BA under FCA.

Shade stress and exogenous 6-BA treatments regulated endogenous hormone homeostasis in wheat flag leaves during both the flowering and grain filling stages. Specifically, IAA and GA contents in wheat flag leaves under BCA were significantly higher than those under FCA, while the ABA and ZT contents were much lower than those under FCA. Further analysis shows that the endogenous IAA, GA and ZT contents in wheat leaves were significantly increased by spraying 6-BA under BCA and FCA compared to not spraying 6-BA, with the most pronounced increase at maturity, while the ABA content of wheat flag leaves was significantly reduced (**Figure 7**).

### Principal component analysis and random forest model

**Figure 8:**
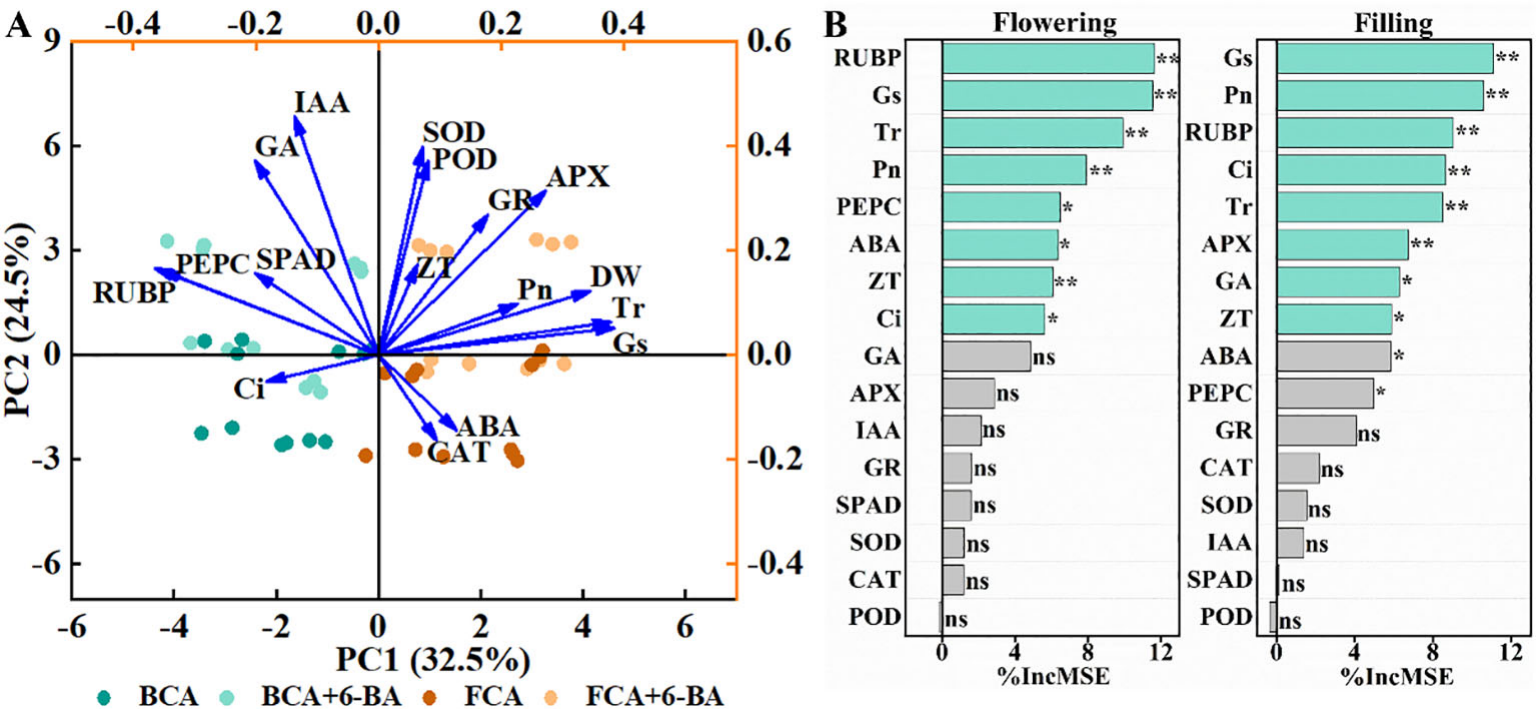
Relationships between physiological indicators and dry matter weight and yield revealed by principal component analysis **(A)** and random forest modelling **(B)**. SOD, superoxide dismutase activity; POD, peroxidase activity; CAT, catalase activity; APX, ascorbate peroxidase activity; GR, glutathione reductase; SPAD, relative chlorophyll content; Pn, net photosynthetic rate; Tr, Transpiration rate; Gs, Stomatal conductance; Ci, intercellular CO_2_ concentration; RuBP, activity of ribulose bisphosphate carboxylase; PEPC, activity of phosphoenolpyruvate carboxylase; ZT, zeatin; ABA, abscisic acid; GA, gibberellin; IAA, indoleacetic acid; DW, dry matter weight. BCA, below canopy area; FCA, far canopy area; BCA+6-BA, exogenous spraying of 6-BA under BCA; FCA+6-BA, exogenous spraying of 6-BA under FCA. * and ** indicate significance at the *P* < 0.05 and 0.01 levels, respectively; ns indicate no significance (*P* > 0.05).

The results of the principal component analysis between the photosynthetic parameters, antioxidant enzymes, hormone content and dry matter accumulation in wheat during the flowering and grain filling stages showed that PC1 and PC2 together explained 57% of the changes in dry matter weight (**Figure 8A**). The main factors influencing dry matter accumulation in wheat were Pn, Tr, Gs, ABA content and APX activity in flag leaves. Furthermore, the random forest model quantified the contributions of photosynthetic and physiological indicators of wheat to mature yield (**Figure 8B**) and the results showed that the activities of RuBPCase and PEPCase, the value of Gs, Tr, Pn and Ci, the content of ABA and ZT of flag leaves during flowering significantly contributed to yield, when the activity of RuBPCase, PEPCase and APX, the value of Gs, Pn, Ci and Tr, the content of GA, ZT, ABA of flag leaves during grain filling stage significantly contributed to yield of maturity.

## Discussion

### Shading inhibits the physiological activity of the flag leaves and thus reduces the wheat yield in the intercropping mode of walnut with wheat

In the fruit-grain intercropping mode of land use optimization, grain crops are disadvantaged in growth, especially for cereal crops such as wheat (Charbonnier et al., 2017; Liu et al., 2020). Similar to the results of Nasielski et al. (2015), who found that soybean growth was significantly inhibited in agroforestry intercropping areas, the present study found a significant decrease in dry matter accumulation and yield of wheat in BCA compared to FCA in a walnut-wheat intercropping pattern in Xinjiang, China (**Figure 3A**; **Table 1**). A highly significant positive correlation was found between dry matter weight and yield at flowering and filling stages (**Figure 2C**). Photosynthesis provides the material basis for yield formation, with more than 90% of wheat yield derived from photosynthesis (Parry et al., 2011), and flag leaf photosynthesis contributing about 40% of yield (Carmo-Silva et al., 2017). The efficiency and regulation of Rubisco and PEPCase are reasonable strategies to increase photosynthesis and yield (Carmo-Silva et al., 2015). However, despite the increase in RuBPCase and PEPCase activities induced by shade stress, Pn, Tr and Gs were significantly suppressed in wheat flag leaves under BCA (**Figure 4**; **Figure 6**), which may be related to the insufficient CO_2_ supply. Interestingly, this study also found a significant positive correlation between APX and CAT activities and photosynthetic parameters (**Figure 6**). Hussain et al. (2020) showed that shade increased antioxidant enzyme activities in soybean and that the magnitude of the effect was greater in the early than in the late stages of shading. However, in the present study, enzymatic antioxidant systems such as SOD, POD, APX were significantly weakened in wheat flag leaves in the near-canopy area (**Figure 5**), which is connected with the plant adaptation mechanism to shade. Hormones are key physiological indicators that balance plant growth and stress tolerance (Stamm and Kumar, 2010; Xu et al., 2021). Previous studies have shown that post-anthesis shade reduces IAA levels but increases ABA levels in maize kernels (Wang et al., 2020). The study on Rumex palustris showed that flooding and shading not only induced GA production but also enhanced sensitivity to GA (Benschop, 2004). In the present study, shading significantly increased IAA and GA contents and decreased ABA and ZT contents in wheat flag leaves (**Figure 7**). The contribution of ZT and ABA contents to yield was significant in wheat flag leaves at the flowering and filling stages (**Figure 8B**). Thus, walnut tree shading significantly altered flag leaf photosynthesis, antioxidant enzyme and hormone contents, which in turn reduced dry matter accumulation and yield of winter wheat.

### 6-BA alleviates inhibition of antioxidant enzymes and hormonal homeostasis in wheat under shade to reduce yield loss

Exogenous hormones alleviate plant stress and promote plant growth under stress conditions by improving plant physiological metabolism (Panigrahy et al., 2019; Mao et al., 2020; Li et al., 2020, 2023). A series of studies have shown that 6-BA plays an important role in plant resistance to drought, waterlogging, high temperature, salinity and other stresses (Wu et al., 2012; Ghaleh et al., 2020; Hu et al., 2022). In maize, 6-BA application improved light energy uptake and dissipation, carbon assimilation and metabolism, and chloroplast ultrastructure, thereby increasing photosynthetic rate in flooded summer maize (Hu et al., 2022). In this study, exogenous 6-BA significantly increased RuBPCase activity in wheat flag leaves (**Figure 4**), thereby promoting photosystem II efficiency and net photosynthesis in wheat (**Figure 3**). Similar to the results of Pan et al. (2013) in hybrid rice, exogenous 6-BA significantly increased the activities of SOD, POD, CAT and APX in flag leaves at anthesis and filling stages, especially at FCA (**Figure 5**), which also indicates that 6-BA application effectively enhanced the scavenging capacity of leaves under shade for superoxide anion, hydrogen peroxide and hydroxyl radical (Li et al., 2020). The most important steps in photosynthesis and the synthesis of hormones for the defensive classes of plants both take place in the chloroplasts (Lu and Yao, 2018). During the flowering and filling stages, exogenous 6-BA increased the content of IAA, ZT, and GA in wheat flag leaves, while reducing the content of ABA (**Figure 7**). Cytokinin is an essential phytohormone involved in the regulation of photosynthesis (Hudecek et al., 2022). Compared to water spraying, 6-BA application decreased growth hormone content, increased cytokinin content and young spike dry matter, and transported more soluble sugars and sucrose from non-spike organs to young spikes (Li et al., 2019). The main factors influencing dry matter accumulation in wheat were Pn, Tr, Gs, ABA content and APX activity in flag leaves (**Figure 8A**). Importantly, exogenous 6-BA also significantly increased wheat dry matter accumulation in BCA and FCA (**Figure 3**). The flowering to grain-filling stage is a critical period for wheat grain development and filling (Djanaguiraman et al., 2020). Increasing photosynthetic capacity by exogenous 6-BA significantly increases wheat thousand kernel weight, which in turn increases yield (**Table 1**). Overall, exogenous 6-BA facilitated the enhancement of physiological activity and photosynthesis in wheat, which in turn partially mitigated the detrimental effects of shading on wheat yield development. 6-BA is also a potentially valuable chemical control regulator that is expected to be beneficial in intensive agriculture.

## Conclusions

When walnut and wheat are intercropped, walnut shading stress significantly reduced antioxidant enzyme activity, ABA and ZT content, and photosynthesis in wheat flag leaves during the flowering and filling stages, thereby reducing dry matter accumulation and mature yield. Under shade stress, exogenous 6-BA significantly increased flag leaf ZT, IAA and GA levels, antioxidant enzymes and photosynthesis, thereby increasing dry matter and yield. The random forest model indicated that under 6-BA and shading treatments, Pn, Gs, Ci, RuBPCase and PEPCase activities, ABA and ZT contents of flag leaves were significant factors influencing wheat yield formation. In conclusion, cytokinins regulate the inhibitory effects of shading stress on wheat photosynthesis, antioxidant capacity and hormone homeostasis, thereby reducing wheat yield loss. Future research will focus on the mechanism by which 6-BA maintains photosynthetic activity in maize leaves and alleviates photoinhibition under shading stress.

## Data Availability

The original contributions presented in the study are included in the article/supplementary material. Further inquiries can be directed to the corresponding author.
